# Complete Genome Sequence of a *Papaya ringspot virus* Isolate from South Korea That Infects *Cucurbita pepo*

**DOI:** 10.1128/genomeA.01289-17

**Published:** 2017-11-30

**Authors:** Dasom Baek, Davaajargal Igori, Seungmo Lim, Un Sun Hwang, Eung Kyoo Choi, Jae Sun Moon

**Affiliations:** a Plant Systems Engineering Research Center, Korea Research Institute of Bioscience and Biotechnology, Daejeon, Republic of Korea; b Biosystems and Bioengineering Program, University of Science and Technology (UST), Daejeon, Republic of Korea; c Jangchun Seed Co., Ltd., Chilgok, Republic of Korea

## Abstract

The complete genome sequence of a *Papaya ringspot virus* (PRSV) isolate from South Korea (SK) infecting squash (*Cucurbita pepo*) was obtained using paired-end RNA sequencing. A BLASTn search of the PRSV SK isolate full-genome sequence showed nucleotide sequence identity ranging from 81% to 83% with previously reported PRSV isolates (GenBank accession numbers KX655874 and EF017707).

## GENOME ANNOUNCEMENT

*Papaya ringspot virus* (PRSV) is a member of the genus *Potyvirus* (family *Potyviridae*). It has a single-stranded positive genome of approximately 10,000 nucleotides (nt) that encodes a large polyprotein (1, 2). PRSV is transmitted by vector aphids in a nonpersistent manner in the field and is also transmissible by mechanical inoculation (1, 3). PRSV is classified into two biotypes, type P (papaya-infecting type) and type W (non-papaya-infecting type), based on the host range, and mainly infects papaya and cucurbits (4, 5). In this study, the complete genome sequence of the PRSV South Korea (SK) isolate infecting *Cucurbita pepo* was obtained.

In May 2014, a *C. pepo* sample showing mosaic symptoms was collected. Paired-end RNA sequencing was performed on an Illumina HiSeq 2500 system at Theragen Bio Institute, Suwon, South Korea. A large single contig of ~10 kb related to PRSV was generated, and eight primer pairs were designed to confirm the sequence. Total RNA was extracted with TRI reagent (Molecular Research Center, Cincinnati, OH, USA), and cDNA was synthesized using the N25 primer. cDNA was amplified by PCR using SuPrime Script PCR premix (GenetBio, Daejeon, South Korea). To determine the 5′ and 3′ terminal sequences, rapid amplification of cDNA ends (RACE) was performed with a 5′/3′ RACE kit (Invitrogen, Carlsbad, CA, USA) according to the manufacturer’s instructions. Amplified DNA was purified and cloned into an RBC T&A cloning vector (RBC Bioscience, Taipei, Taiwan) and sequenced (Macrogen, Daejeon, South Korea). Three clones were sequenced to determine the complete nucleotide sequence.

The PRSV SK isolate consists of 10,324 nt encoding 3,343 amino acids, including an 86-nt 5′ untranslated region (UTR) and a 206-nt 3′ UTR. The PRSV SK isolate shows the highest nucleotide sequence identity with the full genome of the PRSV TM50 isolate (GenBank accession number KX655874) (83%) and the PRSV Andong coat protein (KT884449) (97%). The PRSV SK isolate is therefore highly divergent compared with other PRSV isolates.

### Accession number(s).

The genome sequence of the PRSV SK isolate infecting *Cucurbita pepo* has been deposited in GenBank under the accession number KY996464.
